# Methylone is a rapid-acting neuroplastogen with less off-target activity than MDMA

**DOI:** 10.3389/fnins.2024.1353131

**Published:** 2024-02-07

**Authors:** Jennifer Warner-Schmidt, Martin Stogniew, Blake Mandell, R. Scott Rowland, Eric F. Schmidt, Benjamin Kelmendi

**Affiliations:** ^1^Transcend Therapeutics, New York, NY, United States; ^2^Molecular Synectics, LLC, Jackson, NH, United States; ^3^Laboratory of Molecular Biology, The Rockefeller University, New York, NY, United States; ^4^Department of Psychiatry, Yale School of Medicine, New Haven, CT, United States; ^5^US Department of Veterans Affairs, National Center for PTSD Clinical Neurosciences Division, West Haven, CT, United States

**Keywords:** empathogen, antidepressant, neuroplastogen, PTSD, depression, anxiety

## Abstract

**Background:**

Post-traumatic stress disorder (PTSD) is a highly prevalent psychiatric disorder that can become chronic and debilitating when left untreated. Available pharmacotherapies are limited, take weeks to show modest benefit and remain ineffective for up to 40% of patients. Methylone is currently in clinical development for the treatment of PTSD. Preclinical studies show rapid, robust and long-lasting antidepressant-like and anxiolytic effects. The mechanism of action underlying these effects is not yet fully understood. This study investigated the downstream gene expression changes and signaling pathways affected by methylone in key brain areas linked to PTSD and MDD. It also sought to determine whether neuroplasticity-related genes were involved. We compared effects of methylone with MDMA to explore similarities and differences in their brain effects because MDMA-assisted psychotherapy has recently shown benefit in clinical trials for PTSD and methylone is a structural analog of MDMA.

**Methods:**

Monoamine binding, uptake and release studies were performed and a high-throughput-screen evaluated agonist/antagonist activities at 168 GPCRs *in vitro*. We used RNA sequencing (RNA-seq) to probe drug-induced gene expression changes in the amygdala and frontal cortex, two brain areas responsible for emotional learning that are affected by PTSD and MDD. Rats were treated with methylone or MDMA (both 10 mg/kg, IP), and their responses were compared with controls. We performed functional enrichment analysis to identify which pathways were regulated by methylone and/or MDMA. We confirmed changes in gene expression using immunohistochemistry.

**Results:**

Methylone, a monoamine uptake inhibitor and releaser, demonstrated no off-target effects at 168 GPCRs, unlike MDMA, which showed activity at 5HT2A and 5HT2C receptors. RNA-seq results revealed significant regulation of myelin-related genes in the amygdala, confirmed by immunohistochemistry. In the frontal cortex, methylone significantly upregulated genes implicated in neuroplasticity.

**Conclusion:**

Results suggest that (1) methylone is a rapid-acting neuroplastogen that affects key brain substrates for PTSD and MDD and that (2) methylone appears to exhibit higher specificity and fewer off-target effects than MDMA. Together, these results are consistent with the reported clinical experiences of methylone and MDMA and bolster the potential use of methylone in the treatment of PTSD and, potentially, other neuropsychiatric disorders.

## Introduction

Post-traumatic stress disorder (PTSD) is a debilitating disorder characterized by intrusive flashbacks of a traumatic event, heightened arousal, mood changes, and a high rate of comorbidity with other CNS disorders including major depression (MDD). PTSD has an estimated lifetime prevalence of 6.4–7.8% (Kessler et al., 2005). However, from 2020 to 2022, mental health visits have risen by nearly 40, and 10% of those visits were attributed to patients with PTSD highlighting the urgent need for rapid-acting, effective, and long-lasting treatments for an increasing number of patients (Cantor et al., 2023). Currently, the only Food and Drug Administration (FDA)-approved pharmacotherapies for PTSD are two selective serotonin reuptake inhibitors (SSRIs), sertraline and paroxetine, that have a therapeutic delay of weeks or months and have shown modest efficacy (Ipser and Stein, 2012).

MDMA-assisted psychotherapy has shown potential for alleviating PTSD symptoms (Mitchell et al., 2021; Mitchell et al., 2023). However, the treatment requires extensive concomitant psychotherapy and integrations sessions, its effects are reduced by prior SSRI exposure, and some patients report feeling anxious, fatigued or depressed for days after MDMA use. Methylone, a structural analog of MDMA, has emerged as a promising new treatment for PTSD. Methylone has been well-tolerated in several human studies (Poyatos et al., 2021, 2022, 2023; Kelmendi et al., 2022; Averill et al., 2023; Jones et al., 2023), does not have hallucinogenic effects in humans (Poyatos et al., 2021) or animal models (Yu et al., 2023, under review), and its potential efficacy has been demonstrated in two clinical case series of patients with PTSD (Kelmendi et al., 2022) and MDD (Averill et al., 2023). Recent data from an open-label study of methylone in 14 PTSD patients showed a 36.2 point reduction in the Clinician-Administered PTSD Scale for DSM-5 (CAPS-5) and no significant adverse events (Jones et al., 2023). Importantly, methylone offers several key advantages over MDMA, including a reduced need for specialized concomitant psychotherapy and the potential for coadministration with SSRIs (Feduccia et al., 2021; Warner-Schmidt et al., 2022; Jones et al., 2023).

We have reported that methylone has rapid-acting, robust and long-lasting antidepressant-like and anxiolytic activity in rodents (Warner-Schmidt et al., 2022). Specifically, rats showed a 95–98% reduction in immobility in the forced swim test (FST) 30 min after a single human equivalent dose of 100–150 mg, and which lasted at least 72 h post-dose. Results were not due to any locomotor stimulating effects, and anxiolytic effects in the Open Field Test were observed on the same timescale. Methylone also showed a rapid and robust improvement in fear extinction in a mouse model of PTSD which was also not attributed to locomotor effects or diminished by previous SSRI exposure (Yu et al., 2023; unpublished results). In addition, methylone has shown significant antidepressant-like effects in stress-induced tests like learned helplessness, social defeat, and the sucrose preference (Li et al., 2024). The mechanism underlying the lasting behavioral effects of methylone is not yet understood.

Antidepressants exert their lasting effects at least in part by increasing neuroplasticity in key brain areas, including the amygdala and prefrontal cortex. For example, chronic daily dosing with SSRIs rescue dendritic spine loss in the prefrontal cortex caused by stress (Bessa et al., 2009; Duman and Duman, 2015). Rapid-acting antidepressants also induce neuroplastic changes, but on a shorter timescale (Licznerski and Duman, 2013). Myelin-plasticity is another type of activity-dependent neuroplasticity that is also affected by stress, fear learning and/or affective behavior (Pan et al., 2020; Xin and Chan, 2020). Recent work correlated increased myelin in the amygdala with PTSD in humans and animal models, suggesting it may even predict an individual’s vulnerability to traumatic stress (Long et al., 2021). Various genes and pathways responsible for neuroplastic changes have been described and include neurotrophic factors like brain-derived neurotrophic factor (BDNF) (Yang et al., 2020) or myelin-related proteins such as myelin-basic protein (MBP) (Long et al., 2021). Therefore, analysis of drug-induced RNA expression can serve as a useful screening tool to probe the neuroplastic effects of novel compounds.

This study aims to understand the molecular mechanisms underlying the long-term behavioral effects of methylone and MDMA, and how differences in their pharmacokinetic properties may account for differences their acute effects. To do so, the pharmacokinetic activities of methylone and MDMA at monoamine transporters and GPCRs and changes in RNA expression in key limbic brain areas were examined to further elucidate potentially clinically important pharmacokinetic and neuroplastic changes. Because these compounds are structurally similar and induce comparable therapeutic effects, we hypothesize that genes changed by both methylone and MDMA reflect therapeutic targets, such as neuroplasticity-related genes, in the amygdala and frontal cortex. Lastly, due to their different acute effects, we hypothesize that methylone and MDMA will also lead to drug-specific alterations in gene expression profiles compared with each other.

## Materials and methods

### Animals

Male Sprague Dawley rats (Envigo) were used for binding, uptake and release assays were kept at Gifford Bioscience, Ltd. (Birmingham, UK). The in-life portion of RNA-seq and immunohistochemistry studies were performed at WuXi Apptec (Cranbury, NY) using male Sprague Dawley rats (Hilltop). For all studies, rats weighed ~200 g at arrival and acclimated for at least 2–3 days before use. Animals were group housed in a light- and temperature-controlled environment (20 to 26°C; 30 to 70% humidity; 12 h light/dark cycle) and had *ad libitum* access to standard rodent chow and water. All animal use and procedures were in accordance with established protocols approved by Gifford Bioscience IACUC and Standard Operating Procedures (SOP) or the WuXi Apptec IACUC committee and SOP and Transcend Therapeutics.

### Binding, uptake and release assays

All studies were performed at Gifford Bioscience, Ltd. (Birmingham, UK) according to standard protocols.

#### Competitive radioligand binding

Radioligands used were: [^3^H] Citalopram (PerkinElmer NET1039250UC); [^3^H] Nisoxetine (PerkinElmer NET1084250UC); [^3^H] WIN35428 (PerkinElmer NET1033250UC). Nonspecific compounds used were Citalopram (Tocris Bioscience 1,427); JHW007 (Tocris Bioscience 4,351); Nomifensine (Abcam ab146004). Test compounds used were methylone (Merck M-140) or MDMA (Merck M-103). Rat brains were dissected and tissue was homogenized in cold lysis buffer, centrifuged at 100xg for 2 min and the supernatant was placed in a fresh tube. Supernatant was centrifuged at 13,000 × g for 10 min at 4°C to re-pellet the cell lysate. The pellet was resuspended in fresh wash buffer (50 mM Tris–HCl; 5 mM MgCl_2_; 5 mM EDTA) and centrifuged a third time. The pellet was then resuspended in wash buffer containing 10% sucrose as a cryoprotectant, divided into aliquots (0.3 mL) and stored at −80°C. A sample of the homogenate was analyzed for protein content using the Sigma® BCA assay. On the day of the assay, the membrane preparation was thawed and the pellet resuspended in final assay buffer. Competition binding assays were carried out in 96-well polypropylene plates in a final volume of 250 μL per well. To each well was added 150 μL membranes, 50 μL of non-specific compound or buffer and 50 μL radioligand solution in buffer. The plate was incubated at 30°C for 90 min with gentle agitation. The incubation was stopped by vacuum filtration onto presoaked (PBS buffer with PEI) GF/C filters using a 96-well FilterMate™ harvester, followed by 5 washes with ice-cold wash buffer. Filters were then dried under a warm air stream, sealed in polyethylene, scintillation cocktail added, and the radioactivity counted in a Wallac® TriLux 1,450 MicroBeta counter. For each concentration of drug, non-specific binding was subtracted from total binding to give specific binding. Data was fitted using the non-linear curve fitting routines in Prism® (Graphpad Software Inc) to determine IC_50._ K_i_ was subsequently calculated using the Cheng-Prusoff equation.

#### Uptake inhibition assay

Test and reference compounds [Methylone (Merck M-140), (±)-MDMA (Merck, M-103), Citalopram (Tocris Bioscience 1,427), JHW007 (Tocris Bioscience 4,351), Nomifensine (Abcam ab146004)] were dissolved in DMSO (10 mM) and stored frozen at −20°C. On the day of the assay, compounds were thawed and diluted with assay buffer to 5 × final maximal assay concentration (e.g., 50 μM for a final assay concentration of 10 μM). Rat brain synaptosomes were isolated from Sprague Dawley rats (200-250 g). Brains were dissected and tissue was added to sucrose buffer (0.32 M), homogenized with a dounce-homogenizer and centrifuged at 100 × g to remove cells and debris. Supernatant was collected and centrifuged 17,000 × g for 10 min at 4°C to pellet the synaptosomes. The pellet was resuspended in fresh assay buffer. Uptake assays were carried out in 96-well plates in a final volume of 250 μL per well. To each well was added 150 μL synaptosomes, 50 μL test or non-specific compound or buffer alone. The plate was incubated at 30°C for 30 min with gentle agitation. 50 μL radiolabeled neurotransmitter ([^3^H] 5-HT (PerkinElmer, NET498001MC); [^3^H] DA (PerkinElmer, NET673250UC); [^3^H] NE (PerkinElmer, NET048250UC)) in buffer was then added to each well to initiate the uptake. The plate was incubated 30°C for a further 5 min with gentle agitation. The incubation was stopped by vacuum filtration onto presoaked (0.1% BSA in wash buffer) GF/C filters using a 96-well FilterMate™ harvester, followed by three washes with ice-cold wash buffer. Filters were then dried under a warm air stream, sealed in polyethylene, scintillation cocktail added and the radioactivity counted in a Wallac® TriLux 1,450 MicroBeta counter. For each concentration of drug, non-specific uptake was subtracted from total uptake to give specific uptake. Data was fitted using the non-linear curve fitting routines in Prism® (Graphpad Software Inc) to determine IC_50_.

#### Release assay

Test compounds and synaptosomes were prepared as described for uptake inhibition assays. Synaptosomes treated with [^3^H] 5-HT, [^3^H] DA or [^3^H] NE were loaded onto filter chambers containing GF/C filters and placed in a superfusion system. Oxygenated Krebs buffer was perfused through the chambers at a rate of 1.5 mL/min at 35°C using either an 8-channel or a 12-channel peristaltic pump. Trapped air bubbles were removed from the filters prior to collecting fractions to ensure an even flow over the synaptosomal bed. After a superfusion period of 45 min, 2 basal fractions were collected followed by 6 fractions following the addition of the test drug. In some instances, the fractions containing the test drug were followed by collection of four additional fractions with high potassium (30 mM) to depolarize the synaptosomes. Fractions were 2 mL each. Following collection, an aliquot of (0.25–0.30 mL) each fraction was transferred to a counting plate. After the addition of scintillation cocktail, radioactivity was counted using a Wallac® TriLux 1,450 MicroBeta counter. Once all fractions had been collected, the filters holding the synaptosomes were removed, dried under a stream of warm air. Scintillation cocktail was added, and the filters counted to determine residual radioactivity. Drug-evoked release of neurotransmitter was calculated by subtracting the average of the two basal fractions (collected prior to the drug addition), from the four fractions collected in the presence of drug. The drug-evoked release was then expressed as a percentage of the basal release. Potassium-evoked release was calculated by subtracting the average of two fractions collected prior to the addition of high KCl buffer from that in the two fractions following addition of high KCl buffer. Potassium-stimulated release was calculated as a percentage of basal release. The drug evoked release as a function of drug concentration plotted and the data fitted. Data was fitted using the non-linear curve fitting routines in Prism® (Graphpad Software Inc).

#### GPCR screen

Studies were performed at Eurofins DiscoverX Corporation (Fremont, CA) using the GPCRmax assay according to the manufacturer’s protocols. To determine whether methylone showed agonist or antagonist activity at GPCRs and to compare with effects of MDMA, methylone (1 or 10 μM) or MDMA (1 or 10 μM) were screened in the GPCRmax high-throughput screen of 168 GPCRs. Activity greater than 30% typically indicates agonist activity and inhibition greater than 50% indicates antagonist activity. This assay uses enzyme fragment complementation with β-galactosidase as the functional reporter. When a GPCR is activated, β-galactosidase is recruited and the reporter is detectable. GPCRmax offers a high-throughput screen of 168 GPCRs for agonist or antagonist activity. Briefly, PathHunter cell lines were propagated, seeded into 384-well microplates and incubated at 37°C. Cells were incubated with sample (methylone or MDMA at 1 μM or 10 μM concentrations or appropriate control compounds) for agonist or antagonist determination based on standard protocols and run in triplicate. Microplates were read following signal generation with a PerkinElmer Envision™ instrument for chemiluminescent signal detection. Compound activity was analyzed using CBIS data analysis suite (ChemInnovation, CA) to determine raw values (RLU). For agonist mode assay, percentage activity was calculated using the following formula: % Activity =100% × (mean RLU of test sample - mean RLU of vehicle control) / (mean MAX control ligand - mean RLU of vehicle control). For antagonist mode assay, percentage inhibition was calculated using the following formula: % Inhibition =100% × (1 - (mean RLU of test sample - mean RLU of vehicle control) / (mean RLU of EC_80_ control - mean RLU of vehicle control)).

#### Docking

All calculations were performed with MOE 2022.02 using the Amber10 force field with born solvation. Docking was used to examine potential ligand binding to receptors with experimentally determined structures. The process to prepare the crystallographic protein structures for modeling work as well as preparing the ligands for docking is described below.

#### Protein preparation

In MOE, the QuickPrep procedure was used to standardize and minimize the protein structure subject to tethers on the receptor to relax any strain in the structure while keeping it close to the experimental coordinates. This procedure also determines the ionization state of both protein sidechains and the ligand. After protein preparation, structures were superposed into a common reference frame to compare the binding modes of different ligands.

#### Ligand preparation

In cases where there were two stereoisomers of a particular drug/ligand, each stereoisomer was treated separately for preparation and docking. Each ligand was first ionized at pH 7 with MOE’s Wash function to produce the dominant species. This was followed by conformational generation using the stochastic search method to ensure complex ring systems were adequately sampled. All generated conformations were used as input for docking. Docking poses and the likelihood of binding were evaluated by a combination of MOE’s GBVI/WSA dG docking score, ligand conformational strain, and similarity to related experimental structures.

### RNA sequencing

Rats were given a single dose of methylone (10 mg/kg IP), MDMA (10 mg/kg, IP) or vehicle 8 h before brains were harvested. Frontal cortex and amygdala were rapidly dissected and immediately frozen. In-life work was conducted at WuXi Apptec (Cranbury, NJ). RNA extraction, library preparation, sequencing and analysis was conducted at Azenta Life Sciences (South Plainfield, NJ, USA) as follows:

#### Extraction

Total RNA was extracted from fresh frozen brain samples (amygdala or frontal cortex) sing Qiagen RNeasy Plus Universal mini kit following manufacturer’s instructions (Qiagen, Hilden, Germany).

#### Library preparation with polyA selection and illumina sequencing

RNA samples were quantified using Qubit 2.0 Fluorometer (Life Technologies, Carlsbad, CA, USA) and RNA integrity was checked using Agilent TapeStation 4,200 (Agilent Technologies, Palo Alto, CA, USA). RNA sequencing libraries were prepared using the NEBNext Ultra RNA Library Prep Kit for Illumina using manufacturer’s instructions (NEB, Ipswich, MA, USA). Briefly, mRNAs were initially enriched with Oligod (T) beads. Enriched mRNAs were fragmented for 15 min at 94°C. First strand and second strand cDNA were subsequently synthesized. cDNA fragments were end repaired and adenylated at 3’ends, and universal adapters were ligated to cDNA fragments, followed by index addition and library enrichment by PCR with limited cycles. The sequencing library was validated on the Agilent TapeStation (Agilent Technologies, Palo Alto, CA, USA), and quantified by using Qubit 2.0 Fluorometer (Invitrogen, Carlsbad, CA) as well as by quantitative PCR (KAPA Biosystems, Wilmington, MA, USA). The sequencing libraries were clustered on 5 lanes of a flowcell. After clustering, the flowcell was loaded on the Illumina instrument (4,000 or equivalent) according to manufacturer’s instructions. The samples were sequenced using a 2 x 150bp Paired End (PE) configuration. Image analysis and base calling were conducted by the Control software. Raw sequence data (.bcl files) generated the sequencer were converted into fastq files and de-multiplexed using Illumina’s bcl2fastq 2.17 software. One mismatch was allowed for index sequence identification.

#### Data analysis

After investigating the quality of the raw data, sequence reads were trimmed to remove possible adapter sequences and nucleotides with poor quality. The trimmed reads were mapped to the reference genome available on ENSEMBL using the STAR aligner v.2.5.2b. The STAR aligner is a splice aligner that detects splice junctions and incorporates them to help align the entire read sequences. BAM files were generated as a result of this step. Unique gene hit counts were calculated by using feature Counts from the Subread package v.1.5.2. Only unique reads that fell within exon regions were counted.

#### Pathway analysis

Pathway analysis was performed on selected gene lists based on a statistical cutoff (0.32 ≥ log2FC < −0.32, padj ≤0.1) using Metascape gene annotation and analysis resource (Zhou et al., 2019).

#### Immunohistochemistry

The in-life portion of the study was conducted at WuXi Apptec (Cranbury, NJ). Sectioning and staining of brains took place at Neuroscience Associates (NSA, Knoxville, TN). Rats were treated with a single dose of methylone (10 mg/kg, IP), MDMA (10 mg/kg, IP), or vehicle and sacrificed 24 h later by transcardial perfusion with phosphate-buffered saline (PBS), followed by 4% paraformaldehyde (PFA). Brains were post-fixed in PFA before shipment to NSA for processing and staining. Hemisected brains were mounted, sectioned and stained using standard protocols with myelin-basic protein primary antibody (MBP SMI-99, 1:5000, Biolegend, San Diego, CA) and anti-mouse biotinylated secondary antibody (BA-2001, Vector Laboratories, Newark, CA). Slides were imaged using a Huron Digital Pathology TissueScope LE120 whole slide scanning system. 20x objective was used with a 0.4um/pixel resolution.

#### Statistical analysis

For all studies except RNA-seq, data are presented as the mean ± SEM. Differences between two groups were determined by unpaired t-test, differences between more than two groups were determined by one-way ANOVA and Fisher’s LSD post-hoc test unless otherwise noted. A *p* value<0.05 indicated statistical significance. All analyses were completed using Graphpad Prism software version 9.3.1 (San Diego, CA).

## Results

### Binding, uptake inhibition and release of monoamines by methylone or MDMA

Competitive radioligand binding studies at the serotonin (5HT), norepinephrine (NE) or dopamine (DA) transporters (SERT, NET, and DAT, respectively), revealed that methylone was a less potent inhibitor of SERT than MDMA, demonstrated by a greater inhibition constant (K_i_) (K_i_ = 4.15 μM vs. 2.62 μM). Methylone showed more potent inhibition of NET (K_i_ = 1.15 μM vs. 0.79 μM) and comparable inhibition of DAT (K_i_ = 5.73 μM vs. 5.11 μM) compared with MDMA.

Next, we assayed effects of methylone or MDMA on uptake inhibition of radiolabeled 5HT, NE, and DA using synaptosomes from rat brain. Synaptosomes treated with methylone had four times greater effect on 5HT reuptake inhibition compared with MDMA treatment (IC_50_ = 0.43 μM vs. 1.8 μM). Methylone and MDMA had comparable effects on uptake inhibition of NE (IC_50_ = 0.13 μM vs. 0.12 μM) and DA (IC_50_ = 2.3 μM vs. 2.5 μM).

**Figure 1:**
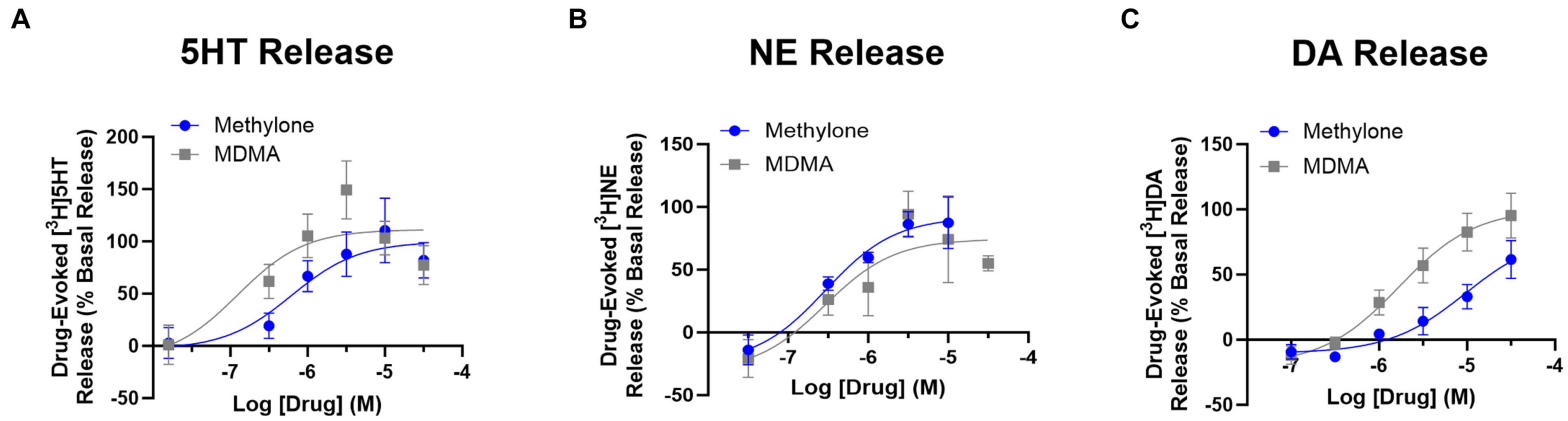
Methylone and MDMA release monoamines. Drug-evoked release of radiolabeled **(A)** 5HT, **(B)** NE or **(C)** DA by methylone or MDMA from rat brain synaptosomes was determined. Data shown are means ± SEM. *N* = 3 per group.

Drug-evoked neurotransmitter release of 5HT, NE or DA was also assayed using rat brain synaptosomes. Less DA was released with methylone treatment compared to MDMA, but similar amounts of 5HT and NE were released with both drugs (Figures 1A-C) (DA effects: drug: *F*_(1,4)_ = 9.932, *p* < 0.05; concentration: *F*_(2,9)_ = 28.66, *p* < 0.0001). The calculated EC_50_ values for neurotransmitter release were: 5HT EC_50_ = 0.62 vs. 0.16 mM; DA EC_50_ = 4.79 vs. 1.42 mM; NE EC_50_ = 0.33 vs. 0.49 mM. Together, these studies confirm that methylone and MDMA are monoamine uptake inhibitors and releasers, but their relative affinities for SERT, NET, and DAT differ.

### Agonist or antagonist activities at GPCRs

**Figure 2:**
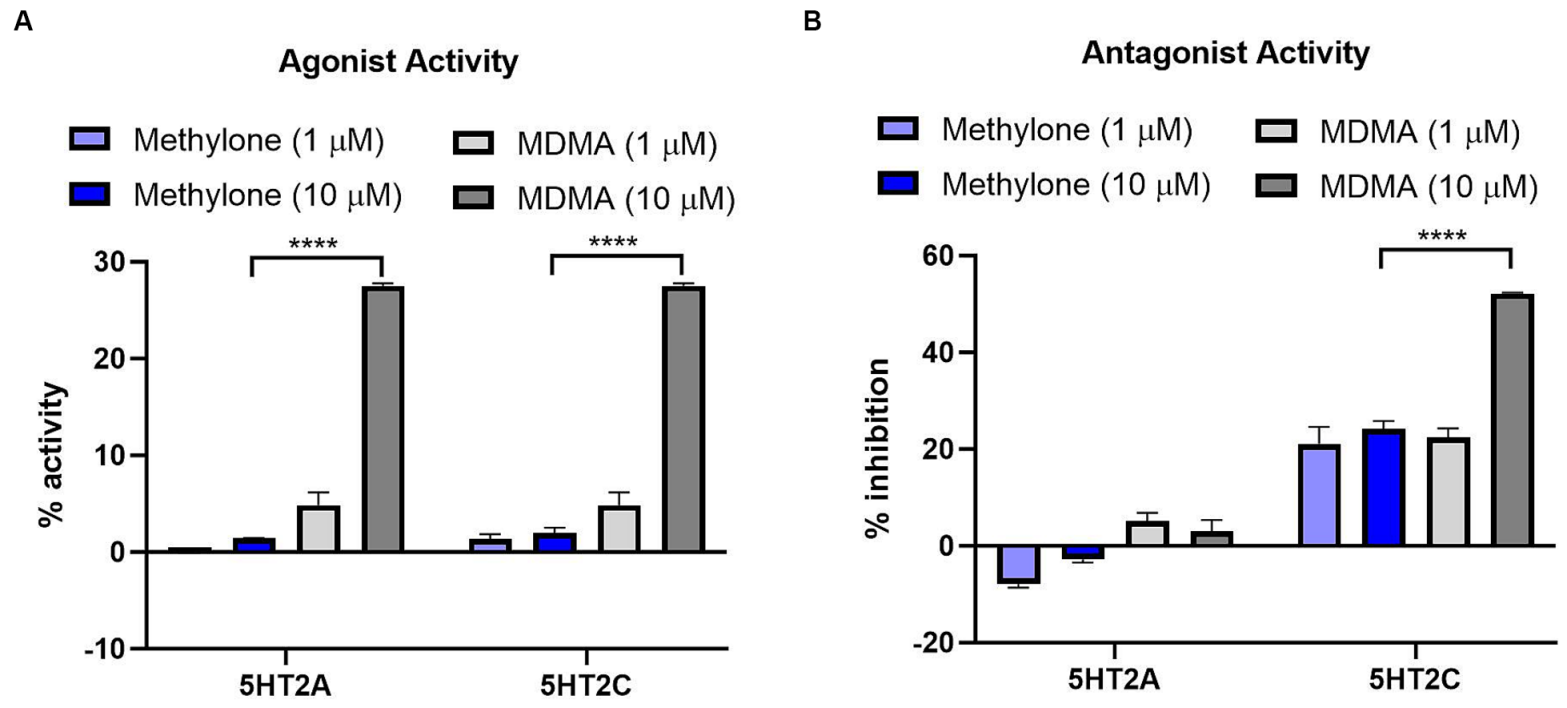
Methylone shows no agonist or antagonist activity at 5HT2A or 5HT2C receptors. Agonist or antagonist activity at GPCRs was determined using the GPCRmax high-throughput screen. Effects of methylone or MDMA (1 or 10 μM) on 5HT2A and 5HT2C receptor **(A)** agonist activity or **(B)** antagonist activity are shown. Data shown are means ± SEM. *N* = 3 replicates per group. ****p < 0.0001 (comparing methylone 10 μM to MDMA 10 μM).

MDMA binds to G-protein coupled receptors (GPCRs), including serotonin receptors (e.g., 5HT2A, 5HT2C) and adrenergic receptors (e.g., α1A, α2A) (Luethi et al., 2019). We used the GPCRmax high throughput screen to examine agonist or antagonist activity on a library of 168 GPCRs. Methylone (1 or 10 μM) had no significant agonist activity at any receptor (Supplementary Table S1). The greatest activity detected was for 5HT5A (13.9%), which was well below the 30% activity threshold of the assay. In contrast, MDMA (10 μM) nearly reached the 30% activity cutoff for agonist activity on 5HT2A and 5HT2C receptors (27.5 and 28.0%, respectively; Figure 2A) which was significantly greater than methylone (5HT2A: *F*_(3,8)_ = 354.8, *p* < 0.0001; 5HT2C: *F*_(3,8)_ = 255.6, p < 0.0001).

Methylone (1 or 10 μM) also had no significant antagonist activity on any GPCR while MDMA showed significant antagonist activity at the 5HT2C receptor (52.2%; Figure 2B) which was statistically different from methylone (5HT2A: *F*_(3,8)_ = 14.57, *p* < 0.01; 5HT2C: *F*_(3,8)_ = 47.64, *p* < 0.0001). Together these data suggest that methylone has no off-target activity at 168 GPCRs whereas MDMA shows some activity at serotonin receptors, consistent with previous reports (Luethi et al., 2019).

### Unlike MDMA, methylone does not dock to 5HT2A or 5HT2C receptors

**Figure 3:**
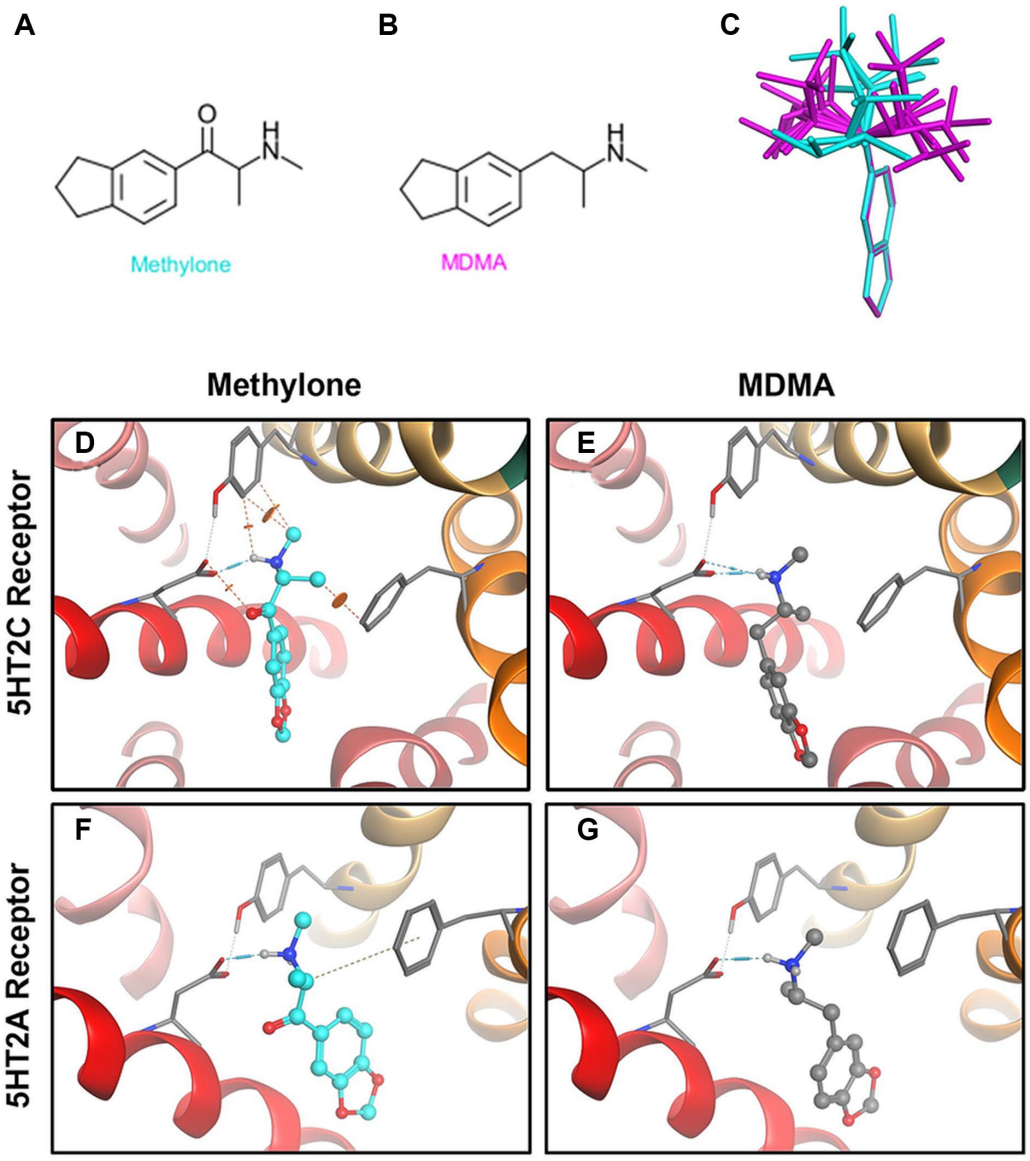
Structural differences between methylone and MDMA support differences in activity at 5HT2A and 5HT2C receptors. Chemical structures of **(A)** methylone or **(B)** MDMA are shown. **(C)** The diagram shows low energy conformations generated in MOE for MDMA (purple) and Methylone (blue) superimposed on the bicyclic ring system. Docking **(D)** methylone (cyan) or **(E)** MDMA (gray) to 5HT2A receptors or **(F,G)** 5HT2C receptors demonstrates that constraints in methylone’s structure predict less binding to receptors. Orange disks indicate steric clashes. Blue cylinders and dashed lines illustrate hydrogen bonds.

Methylone’s chemical structure differs from MDMA only by a ketone group (Figures 3A,B). The diagram (Figure 3C) shows low energy conformations generated for methylone (blue) or MDMA (purple) superimposed on the bicyclic ring system. The ketone in methylone leads to very different shapes with little to no overlap with MDMA. Given subtle differences in receptor binding pocket topography across substrates, the shape difference between MDMA and methylone could lead to very different binding profiles. *In silico* modeling based on the known structures shows that at the 5HT2A receptor, MDMA fits the docking site well but methylone does not fit due to shape differences that cause strain energy (Figures 3D,E). At the 5HT2C receptor, which has a smaller binding pocket than 5HT2A, MDMA fits (Figure 3G). However, due to the very different conformation of methylone, the docking algorithm cannot fit methylone into the binding pocket without generating steric clashes indicated by the orange disks (Figure 3F). This is primarily due to methylone’s different shape and as such, no binding would be expected. The results of the docking study are in alignment with the results from the GPCR screen and show that conformational differences between methylone and MDMA may be a contributing factor to the differences in binding to off-target receptors.

### RNA-seq analysis

**Figure 4:**
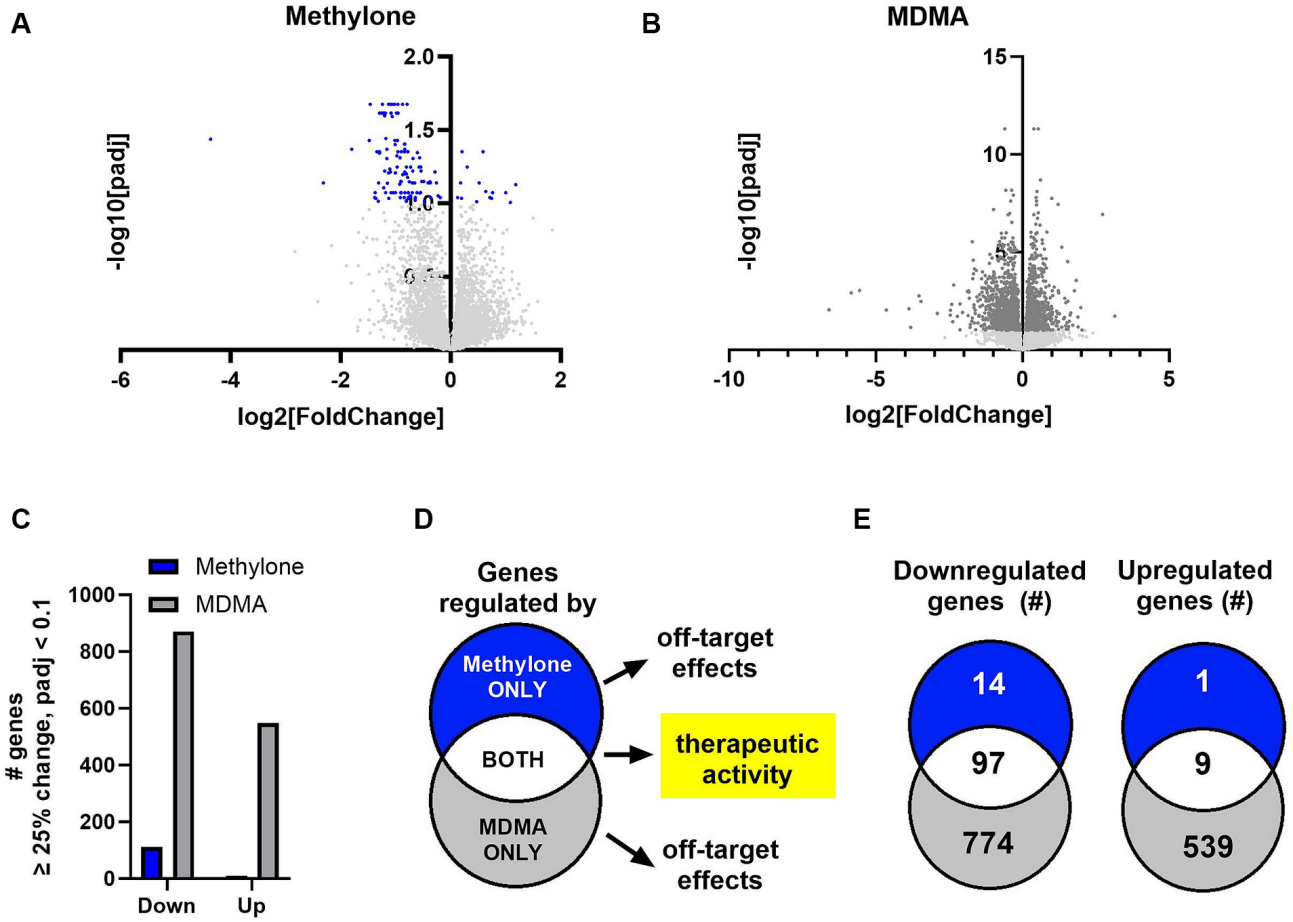
Methylone- and MDMA-induced gene expression changes in the amygdala. Volcano plots show significantly regulated genes in the amygdala after treatment with **(A)** methylone (blue dots) or **(B)** MDMA (dark gray dots) compared to vehicle-injected controls (*N* = 6 per group). Light gray dots represent genes that were not significantly changed by either treatment. **(C)** The number of significantly down- or upregulated genes was quantified. **(D)** An illustration of our hypothesis that genes regulated by both methylone and MDMA are linked to potential therapeutic activity and that genes regulated by methylone or MDMA only are drug-specific and reflect potential off-target effects. **(E)** Venn diagrams illustrating the number of genes significantly regulated by methylone (blue), MDMA (gray) or both (white) reveal that 87% of genes regulated by methylone are also regulated by MDMA, but that only 7% of genes regulated by MDMA are also regulated by methylone.

The amygdala plays a central role in the brain’s response to stress and is affected by CNS disorders like PTSD, MDD and anxiety (Price, 2003; Ressler, 2010; Wang et al., 2019). In order to shed light on methylone’s mechanism of action, we used transcriptomics to determine the impact of methylone or MDMA on the amygdala, specifically to identify which genes and pathways were regulated within hours after a single dose. Following methylone, MDMA, or vehicle treatment, the amygdala and frontal cortex were dissected, and region-specific mRNAs were extracted and analyzed by RNA-seq. Differential expression (DE) analysis of RNA-seq data revealed genes significantly regulated by methylone (Figure 4A) or MDMA (Figure 4B) in the amygdala compared to vehicle-injected controls.

The number of genes significantly up- or downregulated by methylone or MDMA compared to vehicle were quantified. Significantly more gene expression changes were induced by MDMA in amygdala than methylone compared to vehicle controls (Figure 4C). Both methylone and MDMA improve fear extinction, which is thought to underlie potential therapeutic benefit in PTSD (Young et al., 2015; Feduccia and Mithoefer, 2018; Yu et al., 2023). Therefore, we hypothesized that genes commonly changed by both methylone and MDMA reflected those linked to therapeutic activity while drug-specific transcriptional changes underlied drug-specific and potentially off-target effects (Figure 4D). Quantification of the number of significantly regulated genes revealed that nearly all the genes significantly regulated by methylone were also regulated by MDMA. However, 1,313 additional genes were significantly regulated by MDMA only compared to vehicle (Figure 4E).

**Figure 5:**
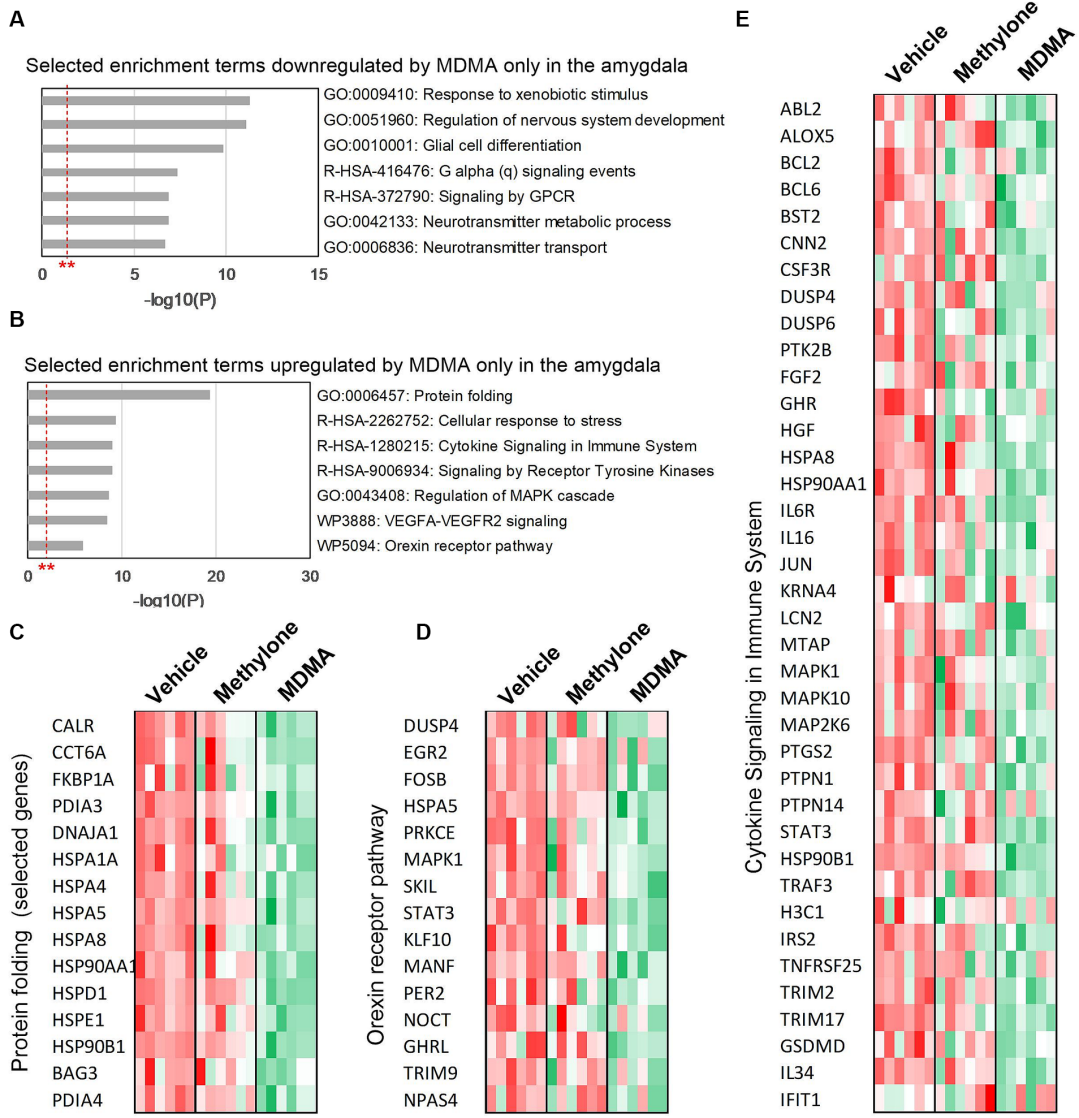
Functional enrichment analysis of genes regulated only by MDMA in the amygdala. Selected enrichment terms from genes that were **(A)** downregulated or **(B)** upregulated by MDMA only are shown. All enrichment terms shown were highly statistically significant. Red dashed line with ** marks the -log(P) = 1 value where *p* = 0.01. Heatmaps generated from terms in **(B)** confirm that MDMA regulates **(C)** protein folding, **(D)** the orexin receptor pathway and **(E)** cytokine signaling, and that these changes are all specific to MDMA and not regulated by methylone.

To classify the list of MDMA-regulated genes and associate them with a particular phenotype, pathway or function, functional enrichment analysis was performed on the 774 downregulated (Figure 5A) and 539 upregulated genes (Figure 5B) in MDMA-treated animals. Among the significantly enriched terms were ‘G alpha (q) signaling events’ and ‘Signaling by GPCR’, consistent with changes in GPCR activity observed (Figure 2A) and previously reported (Luethi et al., 2019). Significantly enriched terms upregulated by MDMA only ‘included receptor tyrosine kinase signaling,’ ‘cellular response to stress,’ ‘protein folding,’ ‘orexin receptor pathway’ and ‘cytokine signaling.’ Heatmaps of genes related to protein folding (Figure 5C), orexin receptor pathway (Figure 5D) or cytokine signaling (Figure 5E) individual animals (*N* = 6 per group) treated with vehicle, methylone or MDMA demonstrated the enrichment of these categories in MDMA-treated animals.

**Figure 6:**
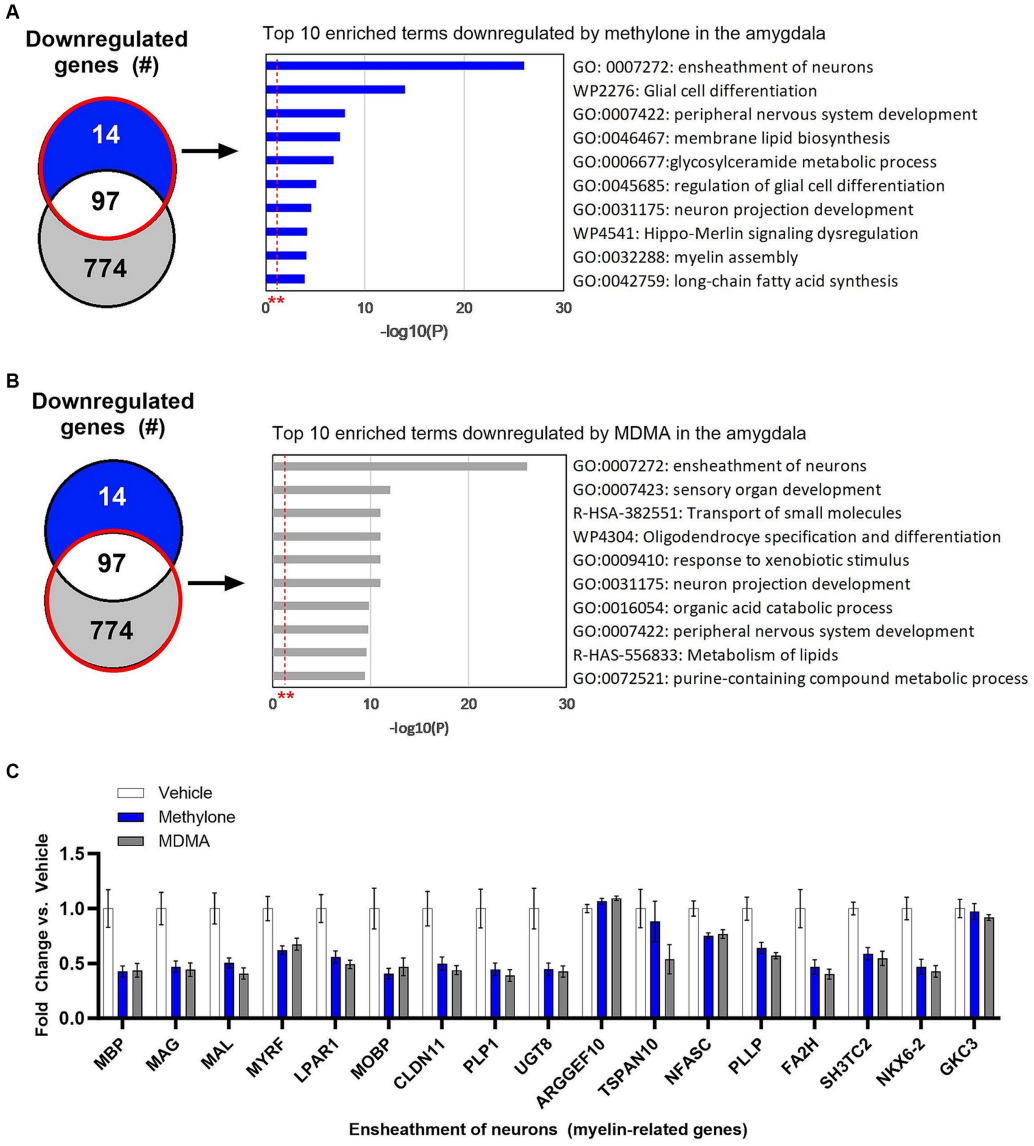
Myelin genes are downregulated in the amygdala after treatment with methylone or MDMA. Functional enrichment analysis of genes downregulated by **(A)** methylone or **(B)** MDMA compared to vehicle controls show highly significant effect on genes related to the ‘ensheathment of neurons.’ All enrichment terms shown were highly statistically significant. Red dashed line with ** marks the -log(P) = 1 value where *p* = 0.01. **(C)** Bar graph shows downregulation of myelin-related genes by methylone and MDMA (*N* = 6 per group).

Methylone treatment did not significantly upregulate many genes in the amygdala (Figure 4E), so functional enrichment analysis focused only on downregulated genes by methylone (Figure 6A) or MDMA (Figure 6B). The most highly significantly enriched term was ‘Ensheathment of neurons’, reflected by the significant downregulation of myelin-associated genes by both methylone and MDMA (Figure 6C). Nearly 20% of all down-regulated genes were related to myelin.

**Figure 7:**
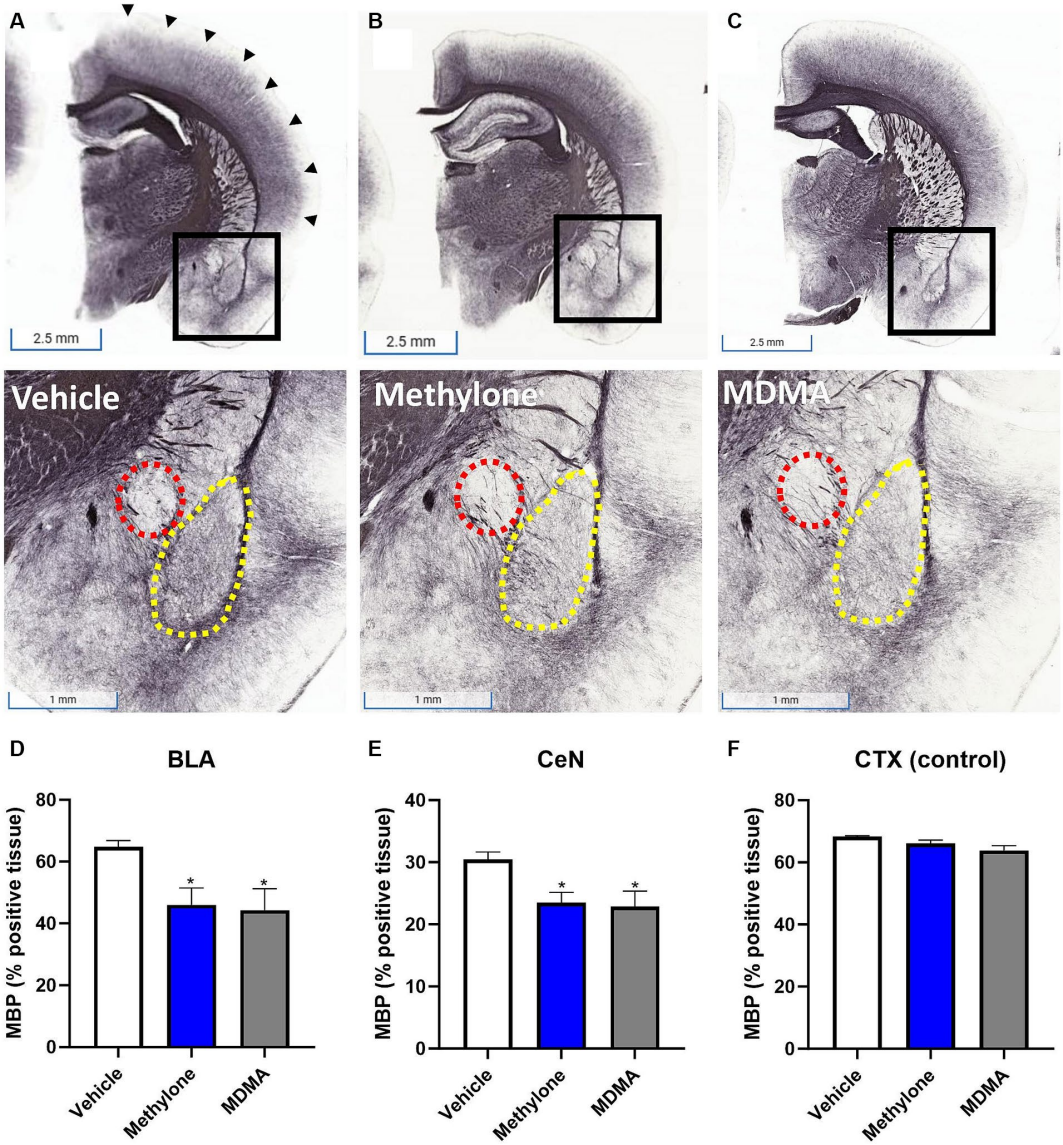
Rapid induction of myelin plasticity by methylone and MDMA and the amygdala. Representative images from immunohistochemical detection of myelin basic protein (MBP) in **(A)** vehicle, **(B)** methylone or **(C)** MDMA-treated rats are shown. Areas in black boxes are magnified below. Quantification of MBP expression (% positive tissue) in the amygdala **(D)** basolateral nucleus (BLA, yellow dashed line), **(E)** central nucleus (CeN, red dashed line) or **(F)** a control region (the cortex, black arrows) is shown. Data are presented are means ± SEM. *p < 0.05 vs. vehicle, *N* = 5–6 per group.

To confirm the results from functional enrichment analysis, levels of myelin basic protein (MBP) were assayed by immunohistochemistry in the amygdala of rats receiving methylone (10 mg/kg, IP), MDMA (10 mg/kg, IP), or vehicle (Figures 7A–C). Results demonstrated a significant reduction in MBP in the basolateral (Figure 7D, *F*_(2,13)_ = 4.417, *p* < 0.05) and central nuclei of the amygdala (Figure 7E, *F*_(2,13)_ = 4.062, *p* < 0.05) compared to vehicle, confirming the RNA-seq results. No change in MBP was observed in the cortex, showing the specificity of this effect in the amygdala (Figure 7F, *F*_(2,13)_ = 3.776, n.s.).

Our results also revealed that the most highly significantly downregulated gene by methylone was Ankyrin Repeat and Kinase Domain Containing 1 (Ankk1), which regulates dopamine synthesis and has been associated with PTSD susceptibility (Niu et al., 2023).

**Figure 8:**
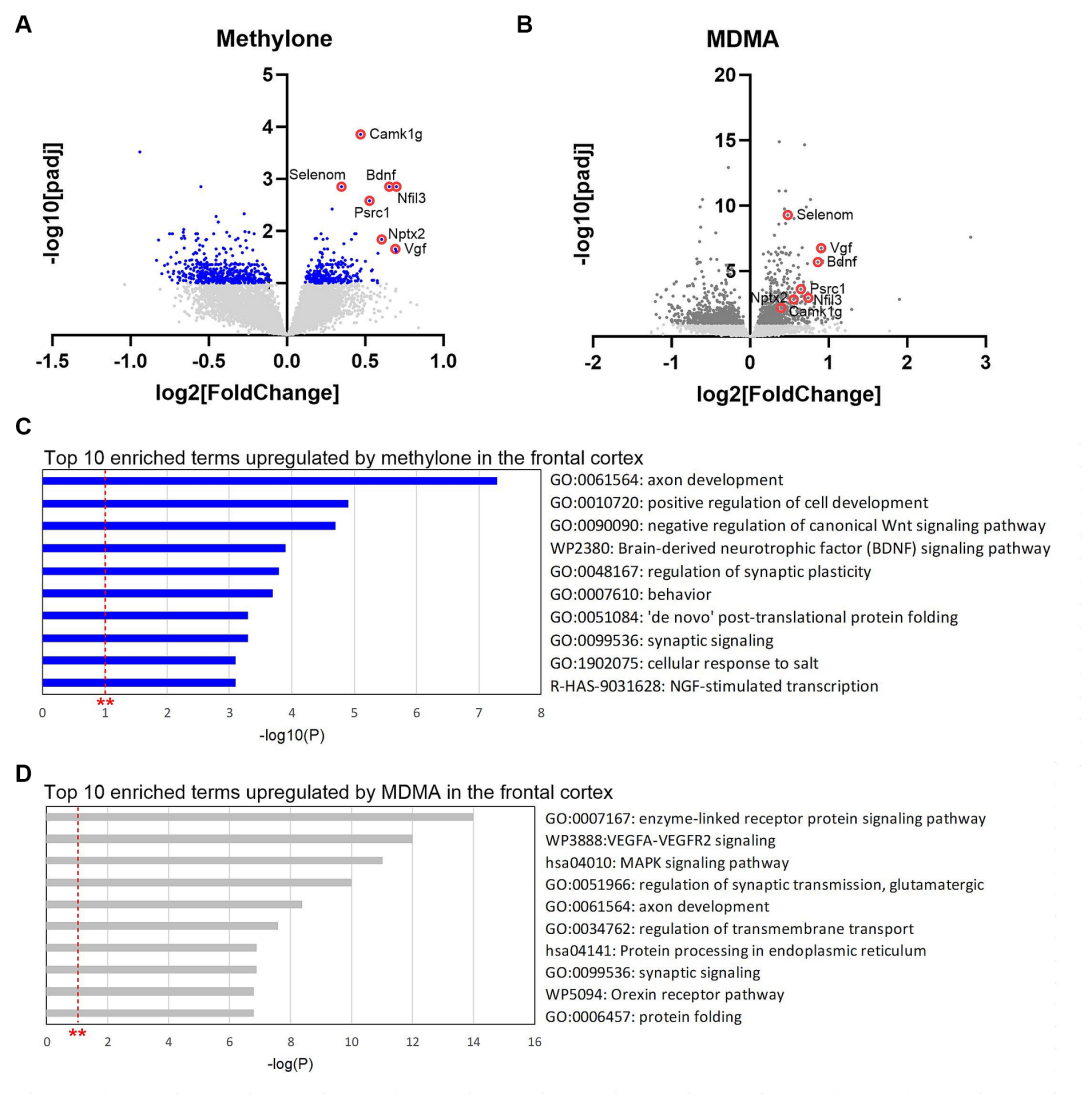
Gene changes in the frontal cortex suggest rapid-acting neuroplasticity. Volcano plots show significantly regulated genes in the frontal cortex by **(A)** methylone (blue dots) or **(B)** MDMA compared to vehicle-injected controls (*N* = 6 per group). Light gray dots represent genes that were not significantly changed by either treatment. Red circles highlight neuroplasticity-related genes discussed in the results section. **(C)** The top 10 enrichment terms in the frontal cortex upregulated by **(C)** methylone or **(D)** MDMA are shown. All enrichment terms shown were highly statistically significant. Red dashed line with ** marks the -log(P) = 1 value where *p* = 0.01.

DE analyses of RNA-seq data from frontal cortex identified transcripts significantly regulated by methylone (Figure 8A) or MDMA in this region (Figure 8B). Consistent with results obtained in the amygdala, ~72% more genes were significantly regulated by MDMA compared to methylone (825 vs. 480 genes), while 154 genes were commonly regulated by both treatments. The top 10 most significant enrichment terms upregulated by methylone included almost exclusively terms linked to synaptic plasticity, cytogenesis and survival, and neurotrophin signaling (e.g., BDNF signaling pathway) (Figure 8C). In addition to BDNF, among the most significantly upregulated genes by methylone were Vgf (non-acronymic), Calcium/Calmodulin-dependent kinase I g (Camk1g), selenoprotein N (Selenom), nuclear factor interleukin 3 related (Nfil3), proline and serine rich coiled-coil 1 (Psrc1) and neuronal pentraxin-2 (Nptx2), all of which are linked to neuroplasticity. MDMA also regulated neuroplasticity genes (Figure 8B), but the top 10 enriched terms differed slightly from those of methylone. Specifically, VEGFA-VEGFR2 signaling, MAPK signaling, protein processing in the endoplasmic reticulum, and protein folding were significantly upregulated (Figure 8D). Orexin receptor pathway and protein folding were also top MDMA enrichment terms in the frontal cortex as observed in the amygdala (Figure 6B, 8D). Together, these results point to changes in synaptic plasticity as a key step in the mechanisms of action of methylone and MDMA and to the specificity of methylone compared with MDMA.

## Discussion

The current study was undertaken to explore the mechanism of action underlying methylone’s rapid, robust, and long-lasting effects as an antidepressant, anxiolytic and treatment for PTSD (Kelmendi et al., 2022; Warner-Schmidt et al., 2022; Averill et al., 2023; Jones et al., 2023; Li et al., 2024). Effects were compared with MDMA to identify similarities and differences between the two compounds that might underlie on- and off-target effects, respectively. Overall, the results from the RNA-seq analysis demonstrated that methylone exhibits a narrower impact on neuroplastic pathways, indicating a more specific mechanism of action compared with MDMA.

Methylone and MDMA are both reuptake inhibitors and releasers of 5HT, NE, and DA. Methylone released comparable levels of NE and 5HT, but less DA than MDMA. Overall, we found that methylone releases NE > 5HT > DA. Previous work suggested that methylone’s effect on monoamine release was NE > DA > 5HT (Eshleman et al., 2013, 2017). This discrepancy could be due to methodological differences. For example, our study was performed in rat brain synaptosomes and the published studies utilized transfected HEK cells. However, recent results using fiber photometry to measure neurotransmitter release *in vivo* are consistent with our results, showing that methylone raises levels of both 5HT and NE in the prefrontal cortex to the same extent as MDMA (Yu et al., 2023). Since dopamine release is involved in impulsivity, emotional sensitivity, and addictive behaviors (Pine et al., 2010), the release of less dopamine by methylone may suggest an advantage over MDMA due to fewer potential off-target effects.

In addition to its effects on monoamine transporters, MDMA has been reported to bind to 5HT, adrenergic and other receptors (Luethi et al., 2019). The results of the current study demonstrate no agonist or antagonist effects of methylone on 168 GPCRs compared with MDMA, which showed activity at 5HT2A and 5HT2C receptors. A docking analysis of binding affinities to 5HT2A and 5HT2C corroborates the results of the GPCR screen, showing that MDMA binds and methylone does not. Differences in the structures of methylone or MDMA could contribute to the observed differences in binding. While the similar overall chemical structures of MDMA and methylone might suggest similar binding characteristics, several important discrepancies can lead to major differences in potential binding to a given receptor. First, methylone contains a ketone carbonyl giving it a hydrogen bond acceptor that is not present in MDMA. This gives important physiochemical differences between the two molecules. For example, methylone has a more polar surface area. Second, the carbonyl in methylone causes significant conformational differences compared with MDMA. Specifically, it is a difference in the torsional energy profile for a ketone to aromatic bond compared to a Csp3 to aromatic bond for MDMA.

Since the hallucinogenic activity of classic psychedelics like psilocybin or LSD have been linked to 5HT2A receptor activation (Lopez-Gimenez and Gonzalez-Maeso, 2018), our findings provide molecular evidence that support the observation that methylone is non-hallucinogenic in animals and humans (Poyatos et al., 2021; Yu et al., 2023, under review). Although MDMA is not a classic hallucinogen, hallucinogenic activity has been reported in some studies (Holze et al., 2020) but not others (Vargas et al., 2021). Insofar as 5HT2A receptor stimulation predicts hallucinogenic activity, our results predict that MDMA has hallucinogenic potential. MDMA also showed activity at the 5HT2C receptor, which modulates the mesolimbic dopamine system (Browne et al., 2017). In animals, the abuse potential of MDMA has been linked to its activity at 5HT2C receptors (Bauer et al., 2015) as well as stimulant effects like increased locomotion (Fletcher et al., 2006). Our results suggest that methylone would have less effect on 5HT2C-mediated behavior.

### A role for myelin plasticity in neuroplastic activity of methylone and MDMA

The amygdala is a key neural substrate for emotional learning whose activity is dysregulated in neuropsychiatric disorders including PTSD, MDD and anxiety (Price, 2003; Ressler, 2010). The amygdala is also a part of neural circuit that has been well-characterized for its involvement in fear conditioning and extinction (Fenster et al., 2018).

The purpose of the RNAseq study was to identify which genes, pathways, and/or functions were commonly regulated by methylone and MDMA, with the hypothesis that they might underlie therapeutic activity. In contrast, genes and pathways regulated by either drug alone might reflect off-target effects. In the amygdala, over a fifth of the transcripts downregulated by both methylone and MDMA encoded myelin-related genes. Stress and antidepressants induce structural and functional changes to brain circuitry (i.e., synaptic plasticity or dendritic remodeling). More recent work shows that adult myelin plasticity is required for proper synaptogenesis, circuit function and learning. Myelin and the oligodendrocytes that produce it play key roles in stress, behavior, and experience-dependent plasticity (Monje, 2018; Pan et al., 2020; Xin and Chan, 2020; Long et al., 2021). Activity dependent changes in myelin, including regulation of oligodendrocytes by BDNF and neurotransmitters, has also been described (Monje, 2018; Xin and Chan, 2020). Increased myelination in the prefrontal cortex correlates with depression (Liu et al., 2022). Most notably, a recent study in humans and animals found that increased myelin in the amygdala was associated with PTSD susceptibility and correlated with fear conditioning in animals (Long et al., 2021). Our results show that methylone and MDMA rapidly reduce myelin in the amygdala, suggesting a mechanism for the rapid-acting effects of these drugs. We speculate that the rapid effect on myelin is a neuroplastic event that makes the aberrant connections that underlie the persistent fear response in PTSD more malleable and facilitates the rewiring of the neural circuit.

### Additional pathways and functions regulated by MDMA only

Genes related to protein folding and degradation, including a number of heat shock proteins (HSPs) were significantly regulated by MDMA and not methylone. HSPs are induced by a variety of environmental stressors including infection and inflammation, but their upregulation might also indicate a mechanism of cytoprotection (Santoro, 2000). More work needs to be done understand the role of these factors in the effects of MDMA.

Orexins are neuropeptides produced in the hypothalamus that have been described to serve several functions depending on the brain area involved. Typically described for their role in wakefulness or response to external stimuli, orexins have been implicated in functions including stress, arousal, vigilance, feeding, reward processing, and drug addiction (Marcus et al., 2001; Aston-Jones, 2005; Winsky-Sommerer et al., 2005; Aston-Jones et al., 2010; Sears et al., 2013). MDMA has been reported to increase energy, reduce appetite and have some potential for addiction, all of which could be mediated by orexins (Baylen and Rosenberg, 2006).

Cytokine signaling was also regulated by MDMA. MDMA has been described previously as an immune system stressor (Connor, 2004). Cytokines can mediate depression and anxiety behaviors (Hu et al., 2022) and may even play a role in the addictive potential of stimulants (Bravo et al., 2023). Notably, no such changes were observed with methylone treatment.

Overall, the effects of methylone on gene expression were more variable between animals than MDMA, which could be due to experimental or biological variability or differences in their neurotransmitter profiles (e.g., less robust dopamine could differentially affect individuals that have more or less sensitivity to dopamine). However, MDMA regulated many more genes, pathways and functions than methylone. These results offer a molecular explanation for the differences between acute effects of methylone and MDMA.

### Rapid-acting neuroplasticity in the frontal cortex

The prefrontal cortex shares strong connectivity with the amygdala and has been described as a critical substrate for fear conditioning and PTSD (Alexandra Kredlow et al., 2022). It is also a substrate of antidepressant effects on neuroplasticity (Moda-Sava et al., 2019).

Two SSRI antidepressants are the only FDA approved pharmacotherapies for PTSD, suggesting potential commonality in the mechanism underlying treatment of PTSD and MDD. Antidepressants have been well-studied for their effects on neuroplasticity, synaptic remodeling (Moda-Sava et al., 2019) and on neurotrophins like brain-derived neurotrophic factor (BDNF) which mediate neuroplastic effects (Castren and Rantamaki, 2010). However, it takes weeks of daily treatment with antidepressants to induce these neuroplastic changes in the brain, correlating with the observed therapeutic delay. Ketamine, a rapid-acting antidepressant, has been shown to induce a sustained increased in neurotrophins like BDNF within hours also correlating with its therapeutic effects (Deyama and Duman, 2020). MDMA has been shown to induce BDNF expression in the prefrontal cortex and other regions (Mouri et al., 2017), but we are the first to report rapid and robust induction of BDNF by methylone (~60% increase compared with controls). Infusions of BDNF into the prefrontal cortex reduce conditioned fear, even in the absence of extinction training (Peters et al., 2010), suggesting that the rapid induction of BDNF by methylone and MDMA may be a critical step in their mechanism of action.

In addition to BDNF, a variety of other genes linked to neuroplasticity were among the most significantly upregulated genes by methylone, including Vgf, Camk1g, Selenom, Nfil3, Psrc1, and Nptx2. Vgf is a neuropeptide that has been linked to the antidepressant-like effects of exercise (Hunsberger et al., 2007). Camk1g is a neuron-specific glucocorticoid-regulated transcription factor whose activity in the amygdala underlies anxiety-related and fear conditioning behavior (Piechota et al., 2022). More generally, calcium/calmodulin-dependent kinases (CAMKs) are closely tied to synaptic plasticity, learning and memory processes (Bayer and Schulman, 2019). Selenom is reported to affect synaptic plasticity and cognitive function (Lin et al., 2023). Nfil3 is a transcription factor that regulates expression of synaptic plasticity genes and is implicated in the regulation of inhibitory long-term potentiation (iLTP) and depression (iLTD) (Chapman et al., 2022). Psrc1 is a microtubule associated protein that promotes cell growth by stimulating the beta-catenin pathway (Hsieh et al., 2007). Nptx1 is a member of a family of proteins that play a crucial role in homeostatic synaptic plasticity by recruiting post-synaptic receptors into synapses. Nptx1 specifically regulates excitatory synaptic plasticity and is essential for the maintenance of LTP (Chapman et al., 2019; de San et al., 2022). These genes were also regulated by MDMA. Together, these results point to rapid and robust changes in gene expression related to rapid-acting neuroplasticity.

Given the molecular evidence for rapid-acting neuroplasticity following methylone and MDMA administration, future studies will explore the effects of methylone and MDMA on structural and functional neuroplasticity and neurocircuitry that underlie neuropsychiatric disorders.

Together, the results of the current study suggest that (1) methylone is a rapid-acting neuroplastogen, rapidly regulating the expression of key synaptic plasticity genes and neurotrophins in brain areas linked to PTSD, MDD and anxiety and (2) overlapping effects of methylone and MDMA are observed and may underlie their common therapeutic effects, but (3) Methylone shows increased specificity as MDMA regulates additional gene expression changes with distinct functional classification, that may be tied to off-target activity.

## Data availability statement

The data discussed in this publication have been deposited in NCBI’s Gene Expression Omnibus (Warner-Schmidt et al., 2024) and are accessible through GEO Series accession number GSE253280. (https://www.ncbi.nlm.nih.gov/geo/query/acc.cgi?acc=GSE253280).

## Ethics statement

The animal study was approved by IACUC committees at WuXi Apptec, and Gifford Biosciences. The study was conducted in accordance with the local legislation and institutional requirements.

## Author contributions

JW-S: Writing – original draft, Writing – review & editing. MS: Writing – review & editing. BM: Writing – review & editing. RR: Writing – review & editing. ES: Writing – review & editing. BK: Writing – review & editing.

## Glossary

5HT: Serotonin
5HT2A: Serotonin receptor 2A
5HT2C: Serotonin receptor 2C
BDNF: Brain-derived neurotrophic factor
CAPS-5: Clinician-Administered Post-traumatic Stress Disorder Scale For DSM-5
DA: Dopamine
DAT: Dopamine transporter
DSM-5: The Diagnostic and Statistical Manual of Mental Disorders, Fifth Edition
FDA: Food and Drug Administration
FST: Forced Swim Test
GPCR: G-protein coupled receptor
IP: Intraperitoneally
MBP: Myelin basic protein
MDD: Major Depressive Disorder
MDMA: 3,4-Methylenedioxy methamphetamine
NE: Norepinephrine
NET: Norepinephrine transporter
PCR: Polymerase chain reaction
PTSD: Post-traumatic stress disorder
RNA: Ribonucleic acid
RNA-seq: Ribonucleic acid sequencing
SERT: Serotonin transporter
SSRI: Selective serotonin reuptake inhibitor
